# Cohort profile: Creation of an e-cohort to Address the Evaluation of Population Secondary Prevention Quality and Outcomes Post-Stroke (ESP-QOPS) in Wales

**DOI:** 10.23889/ijpds.v11i1.3113

**Published:** 2026-04-21

**Authors:** Daniel King, Elizabeth A. Ellins, Hywel T. Evans, Nicholas R. Evans, Mike Gravenor, Julian Halcox, Jonathan Hewitt, Manju Krishnan, Barry McDonnell, Keeron Stone, Daniel E. Harris, Ashley Akbari

**Affiliations:** 1 Swansea University Medical School, Faculty of Medicine, Health and Life Science, Swansea University, Singleton Park, Swansea, UK, SA2 8PP; 2 Department of Clinical Neurosciences, University of Cambridge, Cambridge, UK, CB2 0QQ; 3 Tritech Institute, Hywel Dda University Health Board, Llanelli, UK, SA14 9TD; 4 Pharmacy Department, Hywel Dda University Health Board, Prince Phillip Hospital, Llanelli, UK, SA14 8QF; 5 Swansea Bay University Health Board, Morriston Hospital, Swansea, UK, SA6 6NL; 6 Centre for Cardiovascular Research, Innovation and Development (CURIAD), Cardiff Metropolitan University, Llandaff Campus, Western Avenue, Cardiff, UK, CF5 2YB; 7 National Cardiovascular Research Network (NCRN), Wales; 8 Department of Population Medicine, Cardiff University, Cardiff, UK, CF10 3AT; † Joint senior authors

**Keywords:** stroke, cardiovascular disease, secondary prevention, population health

## Abstract

**Purpose:**

Secondary prevention of stroke requires effective cardiovascular risk factor (RF) management, including blood pressure control, lipid lowering treatment and antithrombotic therapy to reduce the risk of recurrent stroke. Optimal management of risk factors is challenging in older, frailer people, who also have worse outcomes after a stroke. There is limited evidence evaluating secondary prevention quality and outcomes in frail populations. To understand the trends (and factors associated with inequity) in the provision of RF management we developed the ‘Evaluation of Population Secondary Prevention Quality and Outcomes Post-Stroke (ESP-QOPS) in Wales’ e-cohort. The aims of this project include describing (i) the provision of post-stroke evidence-based risk factor management (ii) trends in frailty and outcomes, and (iii) evaluating the impact of suboptimal risk factor provision on adverse outcomes stratified by frailty status.

**Participants:**

We identified 71,696 patients with a first recorded stroke diagnosis in primary or secondary care between 2010-2023 in Wales.

**Findings to Date:**

The incidence of stroke gradually increased from 4,968 to 5,391 patients per year between 2010-2023, with 70% of diagnoses made in the secondary care setting. Of diagnoses made in primary care, less than 20% were subsequently admitted to secondary care.

## Introduction

Lipid lowering, treatment of blood pressure, diabetes and prescribing of antithrombotic therapy (in ischaemic stroke) are prognostically beneficial, evidence-based and guideline endorsed risk management strategies for pharmacological secondary prevention post-stroke [1–6]. However, multiple reports indicate that preventative strategies are often sub-optimally delivered in patients who have survived a stroke [7–9]. Evaluating the quality of cardiovascular risk factor management post-stroke and the factors which optimise or hinder risk management will support the development of targeted, evidence-based strategies to improve care. Frailty is associated with worse outcomes post-stroke, but the influence of frailty on the delivery of risk factor management is not well documented, nor is its impact on outcomes well understood in frail patients [10].

The Evaluation of Population Secondary Prevention Quality and Outcomes Post-Stroke (ESP-QOPS) in Wales project aims to address three work packages (WPs), namely to (WP1) assess the extent of the implementation of secondary prevention risk factor management (i.e., antithrombotic, blood pressure and lipid management) after first stroke; (WP2) identify the prevalence of frailty in adult patients with stroke, comparing clinical outcomes (including mortality) in individuals affected by stroke and concurrent frailty to non-frail individuals with stroke; and (WP3) evaluate the incremental impact of suboptimal implementation of secondary prevention medications on adverse events and all-cause mortality following a first stroke, stratified by frailty status.

This paper describes the protocol for curating a cohort to support the aims of the ESP-QOPS project, as well as the composition of the cohort of interest, including its health, demographic, socioeconomic, and geographic characteristics.

## Methods

### Study Design

An observational cohort study using linked anonymised population-scale, individual-level electronic health record (EHR) data sources for patients with a first-documented stroke event in Wales, United Kingdom, between 2010 and 2023, using the Secure Anonymised Information Linkage (SAIL) Databank [11, 12]. The study period included a look back extending to 2000 to identify prior recorded diagnoses of stroke. General data quality and completeness decrease before 2000, pre-dating the operationalisation of EHR systems and data capture prior to this, which impacted the identification of diagnoses recorded before the year 2000. The lookback period was extended prior to 2000 for all patients identified within our cohort period across both primary and secondary care data sources, where data is available pre-dating 2000.

### Data Sources

The following data sources within the SAIL Databank were linked to support the cohort creation:

- Patient Episode Database for Wales (PEDW), which records hospital admission and discharge dates, diagnoses and operational procedures for all secondary care facilities in Wales.
- Welsh Longitudinal General Practice (WLGP) data, which contain demographic, clinical, and prescribing information for approximately 85% of primary care practices across Wales.
- Welsh Demographic Service Dataset (WDSD), which contains basic demographic information and history of individuals’ residences in Wales, registration with general practices.
- Office for National Statistics (ONS) Census 2011 and 2021 data sources for further demographic and ethnicity characteristics.
- Annual District Death Extract (ADDE) data containing all recorded deaths in Wales with cause details.

### Study Population

**Figure 1: Inclusion Criteria for the Study Cohort:**
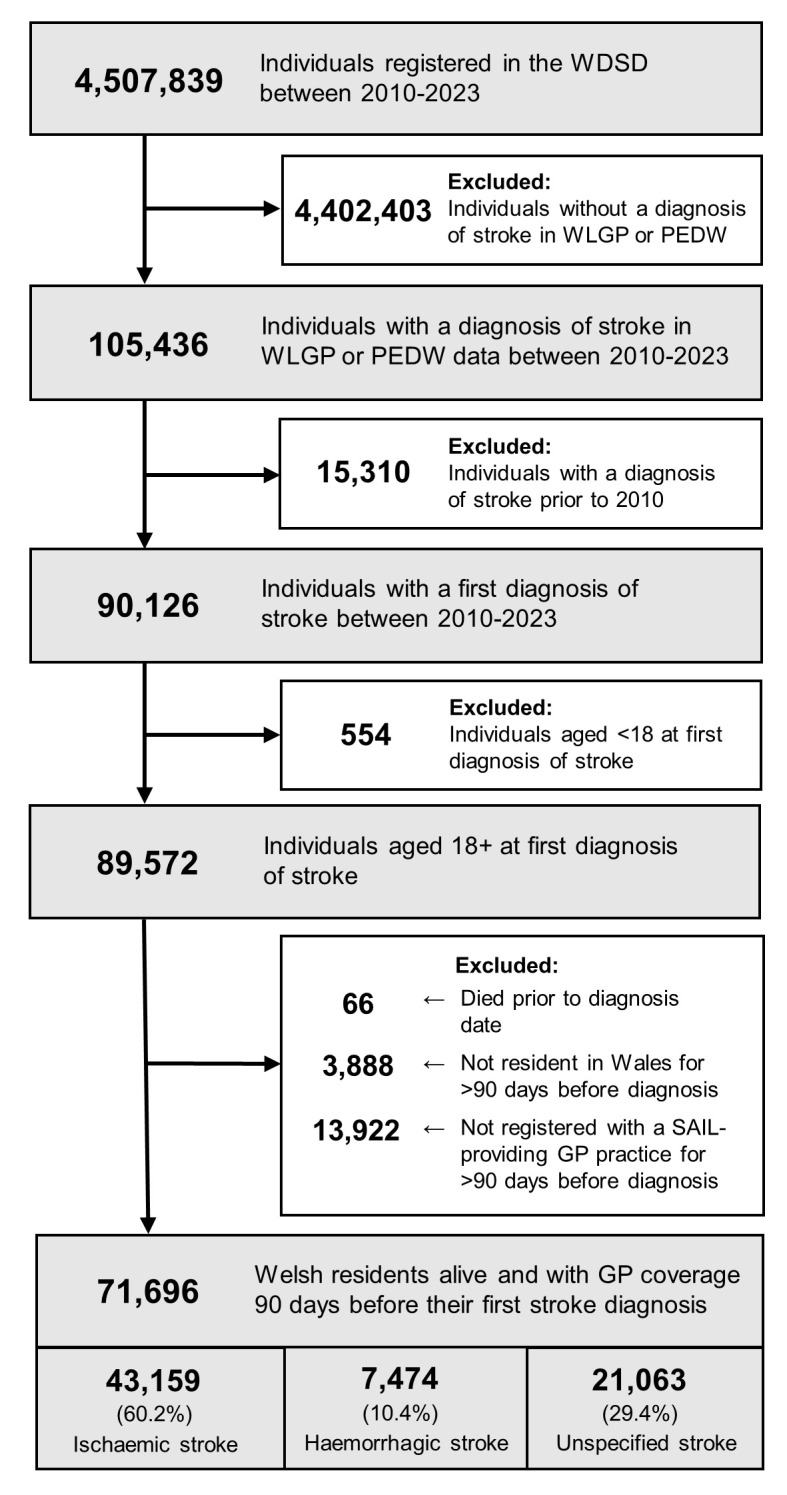


The study population were identified based on the following inclusion criteria: documentation of first stroke diagnosis recorded in primary or secondary care data (see Supplementary Tables 1a and 1b for diagnostic codes) from or following the study start date (1^*st*^ January 2010), aged ≥18 years at diagnosis, resident in Wales and registered with a general practice that provides data to SAIL at diagnosis, with a minimum of 90 days of residency and registration prior to their cohort entry data (Figure 1). The study cohort were subject to the following exclusion criteria: patients with a record of a stroke in Wales prior to study start date and patients without at least one record available in the WLGP data during the study period. These criteria resulted in a cohort representative of 85% of the Welsh population, in line with coverage of the primary care (WLGP) data.

The National Health Service (NHS) in Wales is a publicly funded, free at the point of care health system. There are seven regional health boards (HB) in Wales, providing and/or managing healthcare across both primary and secondary care. Six of the HBs contain regional hyperacute, urgent and rehabilitation stroke units, with patients from the seventh HB accessing their nearest regional facility.

Patients who do not present with acute onset or severe stroke symptoms may also be managed initially in primary care with referral to secondary care outpatient stroke expertise.

Capacity and the ability to respond to the needs of patients may change daily and may be different across each HB. While it is not possible to determine capacity for the provision of stroke diagnosis, especially acute/urgent care using routinely collected health care data we intend to evaluate post-stroke risk factor management to identify gaps (and opportunity) for improving care.

Patients were censored, and length of time in the cohort in days recorded when: they had a mortality date and cause of death record available in the ADDE data source; registration ended with a SAIL providing general practices (i.e., moving practice to a non-SAIL-providing practice for a period of >90 days within the study period); residency in Wales ended (no GP registration for a period of >90 days within the study period).

### Reporting of Incidence

The incidence of stroke disease was reported as the number of patients receiving a first diagnosis of stroke within each year of the observation period. We expressed incidence per 100,000, the denominator being the number of patients resident in Wales, registered with a GP submitting records to SAIL, and aged ≥18 on 1^*st*^ January of the relevant year.

### Covariates and Details of Interest

Demographic characteristics of the cohort of patients were extracted from the WDSD, PEDW, and WLGP data sources throughout each patient’s available history. Characteristics identified from the WDSD data source included the following at cohort entry: age, sex, Lower-layer Super Output Area (LSOA) of residence (version 2011), Welsh Index of Multiple Deprivation (WIMD) quintiles (version 2019), and Rural Urban characteristics.

Time available in the cohort was calculated from stroke diagnosis to the first censor point (see *Study population* above), and the reason for cohort exit recorded. Where mortality was the censor reason, details on the cause of death were captured.

Ethnicity characteristics were extracted using harmonisation of records from all available datasets following the methodology described by Akbari et al. (2024) [13].

Core comorbidities of interest extracted from appropriate primary care (WLGP) and secondary care hospital admission (PEDW) data sources prior to cohort entry included: diabetes, hypertension, dementia, respiratory disease, chronic kidney disease stage 3+, liver disease, smoking status, transient ischaemic attack, significant carotid artery disease, ischaemic heart disease (including records of angina, myocardial infarction, acute coronary syndrome), heart failure, atrial fibrillation, cancer, depression (including recorded diagnosis of depression or symptoms of depression with anti-depressants prescribed within 6-months), stroke diagnosis type (including ischaemic, haemorrhagic, unclassified), prior haemorrhage, obesity.

The catalogue of codes used to describe and categorise comorbidities, medication use and risk factors have been reviewed by a clinical team with extensive experience of undertaking population studies with expert input from clinical coders, cross referenced against previous papers in this field [14–18] and population health statistics. As with all real-world observational studies using anonymised, routinely collected data, it is not possible to account for the accuracy or validity of observations.

### Frailty and Patient Outcomes

Patient frailty will be classified using the electronic Frailty Index (eFI) versions 1 and 2 [19–21] and the Hospital Frailty Risk Score (HFRS) [22]. To support all analyses, frailty will be first calculated at cohort entry (1 day before to ensure that the cohort entry, i.e., the stroke itself, does not contribute to the level of frailty). Further calculations will be derived where data are available (based on the censor point, see above) at 6 months, 1 year, 2 years, and 5 years from cohort entry. Sensitivity and specificity analyses will be conducted on the different frailty indices used to assess validity and generalisability.

To assess post-stroke risk factor management, we will use primary care and pathology data sources to describe the treatment, testing and control of hypertension and lipids, and appropriate antithrombotic treatment prescribing according to guideline standards. Adequate treatment will be defined for each of these categories as follows:

- Blood pressure control - Patients with documented blood pressure below 140/90 mmHg (also 130/90 mmHg to account for historical changes in control levels, below 150/90 mmHg in those over 80 years old).
- Lipid lowering - Documentation of a prescription for lipid lowering therapy post-ischaemic stroke.
- Antithrombotic Therapy - Patients with ischaemic stroke with documentation of prescription for an antiplatelet agent (or documentation of prescription for an anticoagulant agent in those with a diagnosis of atrial fibrillation).

### Patient and Public Involvement

Patient and public involvement (PPI), including experts by experience, was integral to the conception and development of the ESP-QOPS project, including the co-production of its primary objectives, impact, and dissemination protocols. PPI representatives will be continually engaged throughout the project’s life cycle by serving as steering committee members and acting as conduits to the broader stroke community. The project has embedded the Public Involvement in Research Impact Toolkit (PIRIT) at the heart of our PPI development strategy.

## Results

A total of 71,696 patients were identified using this protocol (Table 1). The mean age at stroke diagnosis was 73.0 (±13.9 SD) years and 50.9% were male. Across all years of the study (2010-2023), there was a greater and increasing proportion of patients classified as overweight or obese compared to those under or normal weight (Supplementary Table 2), with a relative increase of 21.7% between 2010-2023.

**Table 1:** Baseline Characteristics at Entry Into the Cohort, Overall and by Year of Diagnosis.

**Characteristic**	**Year**														**Overall**
	**2010**	**2011**	**2012**	**2013**	**2014**	**2015**	**2016**	**2017**	**2018**	**2019**	**2020**	**2021**	**2022**	**2023**	
	n = 4,968	n = 4,858	n = 4,996	n = 4,888	n = 4,951	n = 5,043	n = 5,172	n = 5,172	n = 5,296	n = 5,161	n = 5,140	n = 5,273	n = 5,387	n = 5,391	n = 71,696
**Sex** N (%)															
Male	2,426 (48.8%)	2,358 (48.5%)	2,406 (48.2%)	2,460 (50.3%)	2,481 (50.1%)	2,451 (48.6%)	2,600 (50.3%)	2,647 (51.2%)	2,762 (52.2%)	2,640 (51.2%)	2,731 (53.1%)	2,810 (53.3%)	2,866 (53.2%)	2,883 (53.5%)	36,521 (50.9%)
**Age** Years (standard deviation)															
At first diagnosis	73.8 (13.6)	73.6 (14.0)	73.7 (13.9)	73.6 (13.8)	73.6 (13.9)	73.4 (14.0)	73.1 (14.2)	72.9 (14.2)	73.0 (13.9)	72.5 (14.1)	72.6 (13.8)	72.4 (14.1)	72.1 (13.8)	71.9 (13.8)	73.0 (13.9)
**Weight category** N (%)															
Overweight or Obese*	2,557 (51.5%)	2,560 (52.7%)	2,745 (54.9%)	2,732 (55.9%)	2,834 (57.2%)	2,919 (57.9%)	3,119 (60.3%)	3,131 (60.5%)	3,230 (61%)	3,142 (60.9%)	3,206 (62.4%)	3,309 (62.8%)	3,385 (62.9%)	3,376 (62.7%)	42,245 (58.9%)
**Smoking status** N (%)															
Active smoker*	1,005 (20.2%)	1,071 (22%)	1,037 (20.8%)	1,028 (21%)	1,006 (20.3%)	1,040 (20.6%)	1,088 (21%)	1,087 (21%)	993 (18.8%)	1,078 (20.9%)	1,054 (20.5%)	1,053 (20%)	1,066 (19.8%)	1,093 (20.3%)	14,699 (20.5%)
**Comorbidities** N (%)															
Diabetes	894 (18%)	936 (19.3%)	1045 (20.9%)	988 (20.2%)	1,110 (22.4%)	1,114 (22.1%)	1,155 (22.3%)	1,197 (23.1%)	1,249 (23.6%)	1,225 (23.7%)	1,258 (24.5%)	1,291 (24.5%)	1,332 (24.7%)	1,355 (25.1%)	16,149 (22.5%)
Hypertension	2,833 (57%)	2,764 (56.9%)	2,905 (58.1%)	2,844 (58.2%)	2,859 (57.7%)	2,872 (57%)	2,978 (57.6%)	2,989 (57.8%)	3,070 (58%)	2,883 (55.9%)	2,994 (58.2%)	2,934 (55.6%)	2,984 (55.4%)	3,020 (56%)	40,929 (57.1%)
Atrial Fibrillation	871 (17.5%)	904 (18.6%)	932 (18.7%)	890 (18.2%)	965 (19.5%)	928 (18.4%)	960 (18.6%)	910 (17.6%)	998 (18.8%)	872 (16.9%)	875 (17.0%)	881 (16.7%)	855 (15.9%)	830 (15.4%)	12,671 (17.7%)
Significant Carotid Artery Disease	171 (3.4%)	166 (3.4%)	168 (3.4%)	162 (3.3%)	160 (3.2%)	169 (3.4%)	172 (3.3%)	193 (3.7%)	163 (3.1%)	219 (4.2%)	161 (3.1%)	155 (2.9%)	175 (3.2%)	186 (3.5%)	2,420 (3.4%)

See Supplementary Table 2 for further cohort characteristics of weight, smoking status, among other demographic characteristics. An increase in the presence of diabetes prior to diagnosis was observed, while the presence of hypertension and atrial fibrillation decreased and the proportion of active smokers and those with prior identified significant carotid atherosclerosis remained constant between 2010-23 (Table 1).

**Figure 2: Incidence of stroke according to recorded type, between 2010 and 2023:**
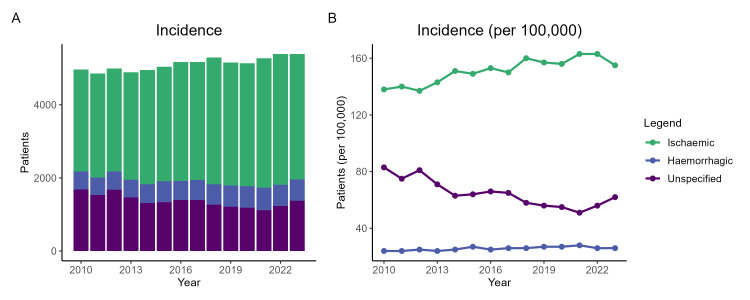
Absolute numbers (A) and per 100,000 of the population (B).

The overall number of patients with a documented stroke increased from 4,968 in 2010 to 5,391 in 2023 (incidence per 100K), primarily driven by an increase in ischaemic stroke, while haemorrhagic stroke increased marginally, and unspecified stroke type decreased between 2010-23 (Figure 2 & Table 1).

When considering the stroke type at first recorded diagnosis, 43,153 (60.2%) of individuals experienced an ischaemic stroke, 7,474 (10.4%) were haemorrhagic, and 21,063 (29.4%) were an unspecified type (Figure 1). However, after allowing for reclassification of the stroke type within the secondary care admission or within seven days of primary care diagnosis, the incidence of unspecified type decreased to 19,180 (26.8%), resulting in an increase in ischaemic stroke to 44,909 (62.6%) and haemorrhagic stroke to 7,607 (10.6%) (Table 2 and Supplementary Figure 1).

**Table 2:** Cohort Baseline Characteristics at Entry into the Study by Stroke Type After Reclassification and Care Setting of Diagnosis.

	**Stroke type**			**Care setting of diagnosis**	
	**Ischaemic**	**Haemorrhagic**	**Unspecified**	**Primary care**	**Secondary care**
	n = 44,909	n = 7,607	n = 19,180	n = 21,305	n = 50,391
**Sex** N (%)					
Male	23,269 (51.8%)	3,869 (50.9%)	9,383 (48.9%)	11,154 (52.4%)	25,367 (50.3%)
**Age** (years) (standard deviation)					
Diagnosis	73.3 (13.6)	70.5 (15.7)	73.1 (14.0)	70.8 (13.8)	73.9 (13.9)
**Weight category** N (%)					
Overweight or Obese*	26,752 (59.6%)	4,032 (53%)	11,461 (59.8%)	12,741 (61.2%)	32,812 (58.4%)
**Smoking status** N (%)					
Active smoker*	9,449 (21%)	1,385 (18.2%)	3,865 (20.2%)	4,670 (21.9%)	10,029 (19.9%)
**Comorbidities** N (%)					
Diabetes	10,611 (23.6%)	1,273 (16.7%)	4,265 (22.2%)	4,558 (21.4%)	11,591 (23%)
Hypertension	26,217 (58.4%)	3,863 (50.8%)	10,849 (56.6%)	11,850 (55.6%)	29,079 (57.7%)
Atrial Fibrillation	8,228 (18.3%)	1,300 (17.1%)	3,143 (16.4%)	3,044 (14.3%)	9,627 (19.1%)
Significant Carotid Artery Disease	2,205 (4.9%)	22 (0.3%)	193 (1%)	2,252 (10.6%)	168 (0.3%)

*See Supplementary Tables 3 and 4 for further cohort characteristics of weight, smoking status, among other demographic characteristics, stratified by stroke type and care setting of diagnosis.

Compared to patients who presented with an ischaemic stroke, those with a haemorrhagic stroke were younger and more likely to be female and actively smoke, while those with unspecified stroke type exhibited similar characteristics to those with ischaemic stroke at baseline, aside from having the greatest proportion of female patients (Table 2 and Supplementary Table 3). The distribution of age at diagnosis was similar among all stroke types and negatively skewed (Figure 3). This distribution was common between sexes, but females were further negatively skewed, indicating females were older than males at first diagnosis of stroke (Figure 3).

**Figure 3: Distribution of age at diagnosis of stroke among the whole population (A), by sex (B) and by stroke type after reclassification (C):**
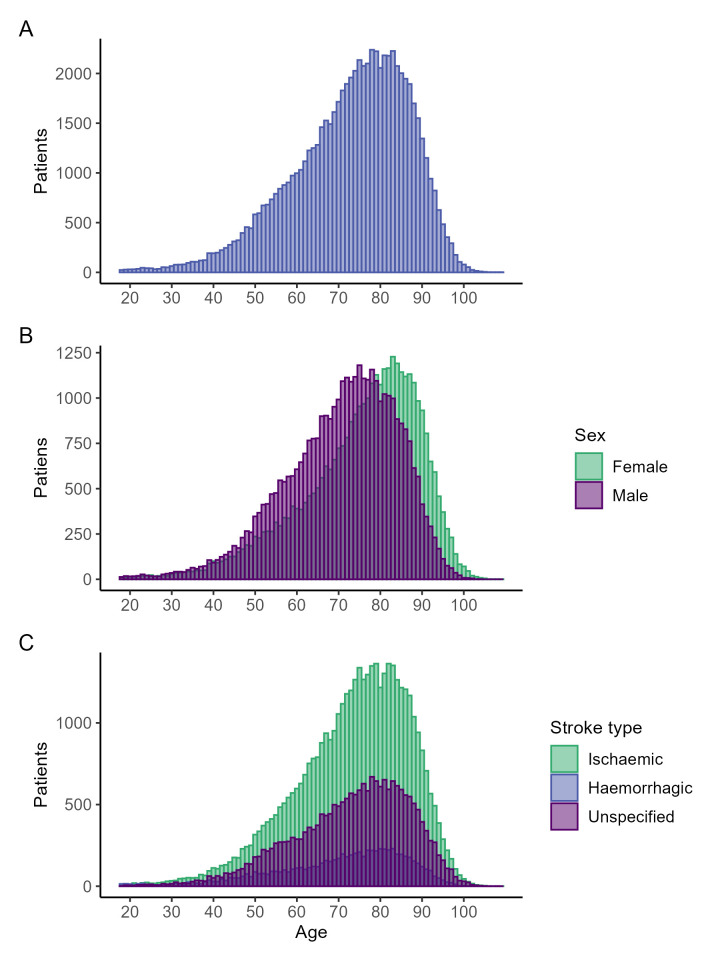


**Figure 4: Patient-healthcare setting interaction pathway following diagnosis to inform risk factor management analyses:**
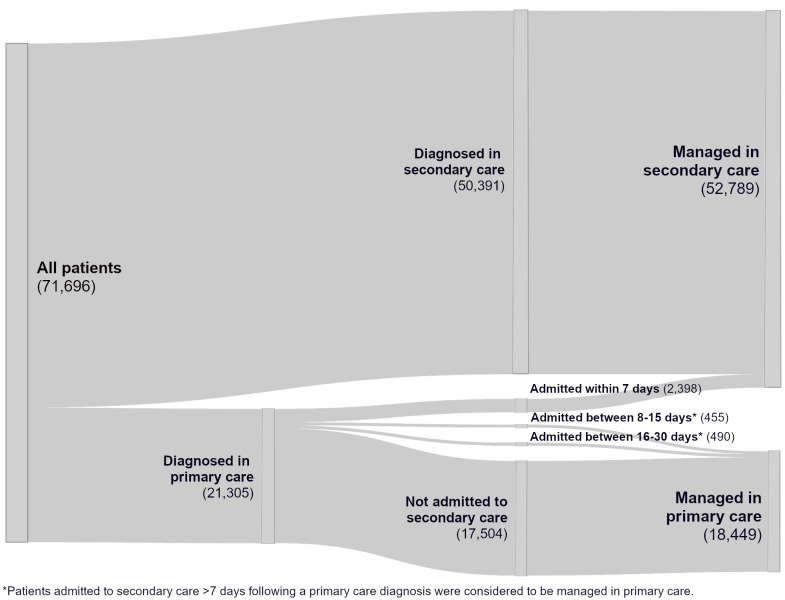


Among the population with a recorded stroke diagnosis during the study period, 21,305 (29.7%) had their first recorded diagnosis in primary care, while 50,391 (70.2%) were first recorded in secondary care (Table 2 and Figure 4). Patients diagnosed in secondary care were older and more likely to be female, have diabetes, hypertension, and atrial fibrillation but less likely to be overweight or obese, actively smoke, and have prior identified significant carotid atherosclerosis than those diagnosed in primary care (Table 2 and Supplementary Table 4).

Among patients with a first recorded stroke in primary care, 82.2% had no subsequent admission to secondary care for stroke within 30 days (Figure 4). Of the 3,343 who were subsequently admitted, 71.7% were admitted within 7 days, 13.6% between 8-15 days, and the remaining 14.7% between 16-30 days. Thus, for analysis of management post-stroke, patients diagnosed in secondary care or admitted within seven days following primary care diagnosis were considered as managed in secondary care (52,789 [73.6%]), while those diagnosed in primary care with admission after seven days or no admission were considered as managed in primary care (18,449 [25.7%]).

## Conclusion

This paper aims to describe the protocol for the curation of a cohort of individuals with a first diagnosis of stroke between 2010-2023 to support the aims of the ESP-QOPS project, and the composition of the cohort of interest. The identified cohort comprised 71,696 stroke patients and described an increasing incidence of stroke from 4,968 to 5,391 patients per year between 2010-23, with 70.2% of these diagnoses made in the secondary care setting.

This cohort will support the aims of the ESP-QOPS project through its use in the delivery of three work packages (WPs). The first work package (WP1) will address the effectiveness of pharmacological secondary prevention care implementation after a first stroke. These analyses will utilise the cohort segmentation described in this protocol to investigate the influence of initial care setting, diagnosis, stroke event type, and other clinical, demographic, socioeconomic, and geographic factors on care delivery, namely antithrombotic, diabetes management, blood pressure control, and lipid management.

WP2 will aim to identify the prevalence of frailty in adult patients with stroke disease and its impact on cardiovascular risk factor management after a first stroke. Frailty characteristics will be used to compare clinical outcomes, including mortality, for individuals affected by stroke and living with frailty compared to those without frailty. Mortality outcomes following diagnosis in each care setting are described in Supplementary Figure 2.

WP3 will aim to derive further insights from the analyses described in Work Packages 1 and 2 through evaluating the incremental impact of suboptimal implementation of secondary prevention medications on recurrent stroke, cardiac events, and all-cause mortality following a first stroke, stratified by frailty status.

## Ethics

Approval for the use of anonymised data in this study, provisioned within the Secure Anonymised Information Linkage (SAIL) Databank, was granted by an independent Information Governance Review Panel (IGRP) under project 1666. The IGRP has a membership comprised of senior representatives from the British Medical Association (BMA), the National Research Ethics Service (NRES), Public Health Wales and Digital Health and Care Wales (DHCW). The usage of additional data was granted by each respective data owner. The SAIL Databank is compliant with General Data Protection Regulations (GDPR) and the UK Data Protection Act.

## Supplementary Files

Supplementary Appendices

## Data Availability

The data used in this study are available in the SAIL Databank at Swansea University, Swansea, UK. All proposals to use SAIL data are subject to review by an independent Information Governance Review Panel (IGRP). Before any data can be accessed, approval must be given by the IGRP. The IGRP gives careful consideration to each project to ensure proper and appropriate use of SAIL data. When access has been approved, it is gained through a privacy-protecting trusted research environment (TRE). SAIL has established an application process to be followed by anyone who would like to access data via https://saildatabank.com/data/apply-to-work-with-the-data. This project was approved by the IGRP (SAIL project number 1666). Participant consent was not required by the IGRP as all data was anonymised prior to the study.

## Licencing

This output is licensed under the Creative Commons Attribution (CC BY) licence and may be shared, adapted, and used commercially, provided appropriate credit is given.
