# Supplementary material for: Cohort profile: Creation of an e-cohort to Address the Evaluation of Population Secondary Prevention Quality and Outcomes Post-Stroke (ESP-QOPS) in Wales
Source: Int J Popul Data Sci. 2026 Apr 21;11(1):3113. doi: 10.23889/ijpds.v11i1.3113 (PMC13213898; doi:10.23889/ijpds.v11i1.3113)
Supplement: Supplementary Appendices [file ijpds-11-3113-s001.pdf]

Table of Contents

Diagnostic codes 2

Supplementary Table 1a. ICD-10 codes for diagnoses of stroke. 2

Supplementary Table 1b. Read (version 2) codes for diagnoses of stroke. 3

Supplementary Table 2. Cohort baseline characteristics at entry into the study, by year of diagnosis, continued. 5

Supplementary Table 3. Cohort baseline characteristics at entry into the study, by stroke type, continued. 9

Supplementary Table 4. Cohort baseline characteristics at entry into the study, by care setting of diagnosis, continued. 11

Supplementary Figure 1. Care setting of diagnosis and reclassification of stroke type following first record 13

Supplementary Figure 2. Care setting of diagnosis and death outcomes in the year following diagnosis. 14

Diagnostic codes

Supplementary Table 1a. ICD-10 codes for diagnoses of stroke.

Description	Code
Intracerebral haemorrhage in hemispheresubcortical	I610
Intracerebral haemorrhage in hemispherecortical	I611
Intracerebral haemorrhage in hemisphereunspecified	I612
Intracerebral haemorrhage in brain stem	I613
Intracerebral haemorrhage in cerebellum	I614
Intracerebral haemorrhageintraventricular	I615
Intracerebral haemorrhagemultiple localized	I616
Other intracerebral haemorrhage	I618
Intracerebral haemorrhageunspecified	I619
Cerebral infarct due to thrombosis of precerebral arteries	I630
Cerebral infarction due to embolism of precerebral arteries	I631
Cereb infarct due unsp occlusion or stenos precerebrl arts	I632
Cerebral infarction due to thrombosis of cerebral arteries	I633
Cerebral infarction due to embolism of cerebral arteries	I634
Cerebrl infarct due unspec occlusion or stenos cerebrl arts	I635
Cereb infarct due cerebral venous thrombosisnonpyogenic	I636
Other cerebral infarction	I638
Cerebral infarction unspecified	I639
Occlusion and stenosis of vertebral artery	I650*
Occlusion and stenosis of basilar artery	I651*
Occlusion and stenosis of carotid artery	I652*
Occlusion and stenosis of multip and bilat precerebrl arts	I653*
Occlusion and stenosis of other precerebral artery	I658*
Occlusion and stenosis of unspecified precerebral artery	I659*
Occlusion and stenosis of middle cerebral artery	I660*
Occlusion and stenosis of anterior cerebral artery	I661*
Occlusion and stenosis of posterior cerebral artery	I662*
Occlusion and stenosis of cerebellar arteries	I663*
Occlusion and stenosis of multiple and bilat cerebrl arts	I664*
Occlusion and stenosis of other cerebral artery	I668*
Occlusion and stenosis of unspecified cerebral artery	I669*
Sequelae of cerebral infarction	I693
Sequelae of intracerebral haemorrhage	I691
Strokenot specified as haemorrhage or infarction	I64X
Sequelae of strokenot spec as haemorrhage or infarction	I694

*I65 and I66 code diagnoses were only valid if followed by an I63 or I64 code within 30 days.

Supplementary Table 1b. Read (version 2) codes for diagnoses of stroke.

Description	Code
Intracerebral haemorrhage	G61..
Cortical haemorrhage	G610.
Internal capsule haemorrhage	G611.
Basal nucleus haemorrhage	G612.
Cerebellar haemorrhage	G613.
Pontine haemorrhage	G614.
Bulbar haemorrhage	G615.
External capsule haemorrhage	G616.
Intracerebral haemorrhage, intraventricular	G617.
Intracerebral haemorrhage, multiple localized	G618.
Lobar cerebral haemorrhage	G619.
Intracerebral haemorrhage in hemisphere, unspecified	G61X.
Left sided intracerebral haemorrhage, unspecified	G61X0
Right sided intracerebral haemorrhage, unspecified	G61X1
Intracerebral haemorrhage NOS	G61z.
Precerebral arterial occlusion	G63..
Basilar artery occlusion	G630.*
Carotid artery occlusion	G631.*
Vertebral artery occlusion	G632.*
Multiple and bilateral precerebral arterial occlusion	G633.*
Carotid artery stenosis	G634.*
Other precerebral artery occlusion	G63y.*
Cerebral infarct due to thrombosis of precerebral arteries	G63y0
Cerebral infarction due to embolism of precerebral arteries	G63y1
Precerebral artery occlusion NOS	G63z.*
Cerebral arterial occlusion	G64..*
Cerebral thrombosis	G640.
Cerebral infarction due to thrombosis of cerebral arteries	G6400
Cerebral embolism	G641.
Cerebral infarction due to embolism of cerebral arteries	G6410
Cerebral infarction NOS	G64z.
Brainstem infarction	G64z0
Wallenberg syndrome	G64z1
Left sided cerebral infarction	G64z2
Right sided cerebral infarction	G64z3
Infarction of basal ganglia	G64z4
Occlusion and stenosis of middle cerebral artery	G6770*
Occlusion and stenosis of anterior cerebral artery	G6771*

Occlusion and stenosis of posterior cerebral artery	G6772*
Occlusion and stenosis of cerebellar arteries	G6773*
Occlusion+stenosis of multiple and bilat cerebral arteries	G6774
Cereb infarct due unsp occlus/stenos precerebr arteries	G6W..
Cerebrl infarctn due/unspcf occlusn or sten/cerebrl artrs	G6X..
Stroke and cerebrovascular accident unspecified	G66..
Brain stem stroke syndrome	G663.
Cerebellar stroke syndrome	G664.
Left sided CVA	G667.
Right sided CVA	G668.
Cerebral palsy, not congenital or infantile, acute	G669.

*Diagnoses were only valid if followed by a non-asterisk code within 30 days.

Supplementary Table 2. Cohort baseline characteristics at entry into the study, by year of diagnosis, continued.

	2010	2011	2012	2013	2014	2015	2016	2017	2018	2019	2020	2021	2022	2023	Overall
	n = 4,968	n = 4,858	n = 4,996	n = 4,888	n = 4,951	n = 5,043	n = 5,172	n = 5,172	n = 5,296	n = 5,161	n = 5,140	n = 5,273	n = 5,387	n = 5,391	n = 71,696
**Sex**															
Male	2,426 (48.8%)	2,358 (48.5%)	2,406 (48.2%)	2,460 (50.3%)	2,481 (50.1%)	2,451 (48.6%)	2,600 (50.3%)	2,647 (51.2%)	2,762 (52.2%)	2,640 (51.2%)	2,731 (53.1%)	2,810 (53.3%)	2,866 (53.2%)	2,883 (53.5%)	36,521 (50.9%)
**Age (years)**															
Diagnosis	73.8 (13.6)	73.6 (14.0)	73.7 (13.9)	73.6 (13.8)	73.6 (13.9)	73.4 (14.0)	73.1 (14.2)	72.9 (14.2)	73.0 (13.9)	72.5 (14.1)	72.6 (13.8)	72.4 (14.1)	72.1 (13.8)	71.9 (13.8)	73.0 (13.9)
**WIMD Quintile**															
1 (Most deprived)	992 (20%)	1,027 (21.1%)	951 (19%)	1,041 (21.3%)	985 (19.9%)	1,004 (19.9%)	980 (18.9%)	1,018 (19.7%)	1,077 (20.3%)	1,058 (20.5%)	1,071 (20.8%)	1,025 (19.4%)	1,036 (19.2%)	1,017 (18.9%)	14,282 (19.9%)
2	1,051 (21.2%)	1,016 (20.9%)	1,073 (21.5%)	1,030 (21.1%)	1,023 (20.7%)	1,124 (22.3%)	1,089 (21.1%)	1,070 (20.7%)	1,105 (20.9%)	1040 (20.2%)	1,081 (21%)	1,049 (19.9%)	1,126 (20.9%)	1,087 (20.2%)	14,964 (20.9%)
3	995 (20%)	1,017 (20.9%)	1,029 (20.6%)	949 (19.4%)	1,018 (20.6%)	1,016 (20.1%)	1,013 (19.6%)	1,038 (20.1%)	1,063 (20.1%)	1,097 (21.3%)	1,015 (19.7%)	1,105 (21%)	1,047 (19.4%)	1,161 (21.5%)	14,563 (20.3%)
4	981 (19.7%)	910 (18.7%)	972 (19.5%)	957 (19.6%)	1,013 (20.5%)	976 (19.4%)	1,019 (19.7%)	1,016 (19.6%)	1,046 (19.8%)	997 (19.3%)	1,000 (19.5%)	1,018 (19.3%)	1,068 (19.8%)	1,095 (20.3%)	14,068 (19.6%)
5 (Least deprived)	949 (19.1%)	888 (18.3%)	971 (19.4%)	911 (18.6%)	912 (18.4%)	923 (18.3%)	1,071 (20.7%)	1,030 (19.9%)	1,005 (19%)	969 (18.8%)	973 (18.9%)	1,076 (20.4%)	1,110 (20.6%)	1,031 (19.1%)	13,819 (19.3%)
**Rural/Urban classification**															

Rural town and fringe	676 (13.6%)	640 (13.2%)	706 (14.1%)	657 (13.4%)	600 (12.1%)	637 (12.6%)	693 (13.4%)	723 (14%)	725 (13.7%)	669 (13%)	682 (13.3%)	733 (13.9%)	714 (13.3%)	764 (14.2%)	9,619 (13.4%)
Rural town and fringe in a sparse setting	201 (4%)	209 (4.3%)	233 (4.7%)	195 (4%)	212 (4.3%)	203 (4%)	231 (4.5%)	216 (4.2%)	198 (3.7%)	193 (3.7%)	188 (3.7%)	213 (4%)	201 (3.7%)	200 (3.7%)	2,893 (4%)
Rural village and dispersed	298 (6%)	295 (6.1%)	286 (5.7%)	313 (6.4%)	297 (6%)	319 (6.3%)	344 (6.7%)	312 (6%)	348 (6.6%)	331 (6.4%)	313 (6.1%)	354 (6.7%)	324 (6%)	306 (5.7%)	4,440 (6.2%)
Rural village and dispersed in a sparse setting	351 (7.1%)	404 (8.3%)	391 (7.8%)	337 (6.9%)	432 (8.7%)	381 (7.6%)	400 (7.7%)	381 (7.4%)	399 (7.5%)	390 (7.6%)	359 (7%)	416 (7.9%)	396 (7.4%)	436 (8.1%)	5,473 (7.6%)
Urban city and town	3,321 (66.8%)	3,212 (66.1%)	3,300 (66.1%)	3,304 (67.6%)	3,302 (66.7%)	3,402 (67.5%)	3,408 (65.9%)	3,434 (66.4%)	3,522 (66.5%)	3,474 (67.3%)	3,487 (67.8%)	3,464 (65.7%)	3,655 (67.8%)	3,589 (66.6%)	47,874 (66.8%)
Urban city and town in a sparse setting	121 (2.4%)	98 (2%)	80 (1.6%)	82 (1.7%)	108 (2.2%)	101 (2%)	96 (1.9%)	106 (2%)	104 (2%)	104 (2%)	111 (2.2%)	93 (1.8%)	97 (1.8%)	96 (1.8%)	1,397 (1.9%)
**Ethnic group**															
Asian, Black, Mixed and other	44 (0.9%)	74 (1.5%)	76 (1.5%)	62 (1.3%)	70 (1.4%)	77 (1.5%)	85 (1.6%)	89 (1.7%)	90 (1.7%)	92 (1.8%)	106 (2.1%)	110 (2.1%)	115 (2.1%)	116 (2.2%)	1,206 (1.7%)
White	4,239 (85.3%)	4,490 (92.4%)	4,723 (94.5%)	4,638 (94.9%)	4,717 (95.3%)	4,802 (95.2%)	4,950 (95.7%)	4,971 (96.1%)	5,094 (96.2%)	4,991 (96.7%)	4,979 (96.9%)	5,136 (97.4%)	5,232 (97.1%)	5,228 (97%)	68,190 (95.1%)
Unknown	685 (13.8%)	294 (6.1%)	197 (3.9%)	188 (3.8%)	164 (3.3%)	164 (3.3%)	137 (2.6%)	112 (2.2%)	112 (2.1%)	78 (1.5%)	55 (1.1%)	27 (0.5%)	40 (0.7%)	47 (0.9%)	2,300 (3.2%)
**Weight category**															
Obese	1,108 (22.3%)	1,179 (24.3%)	1,221 (24.4%)	1,295 (26.5%)	1,304 (26.3%)	1,329 (26.4%)	1,481 (28.6%)	1,501 (29%)	1,571 (29.7%)	1,462 (28.3%)	1,499 (29.2%)	1,571 (29.8%)	1,626 (30.2%)	1,642 (30.5%)	19,789 (27.6%)
Overweight	1,449 (29.2%)	1,381 (28.4%)	1,524 (30.5%)	1,437 (29.4%)	1,530 (30.9%)	1,590 (31.5%)	1,638 (31.7%)	1,630 (31.5%)	1,659 (31.3%)	1,680 (32.6%)	1,707 (33.2%)	1,738 (33%)	1,759 (32.7%)	1,734 (32.2%)	22,456 (31.3%)

Normal weight	1,262 (25.4%)	1,270 (26.1%)	1,303 (26.1%)	1,304 (26.7%)	1,303 (26.3%)	1,300 (25.8%)	1,293 (25%)	1,279 (24.7%)	1,346 (25.4%)	1,335 (25.9%)	1,299 (25.3%)	1,316 (25%)	1,321 (24.5%)	1,359 (25.2%)	18,290 (25.5%)
Underweight	170 (3.4%)	163 (3.4%)	175 (3.5%)	170 (3.5%)	188 (3.8%)	194 (3.8%)	169 (3.3%)	176 (3.4%)	187 (3.5%)	187 (3.6%)	171 (3.3%)	187 (3.5%)	184 (3.4%)	177 (3.3%)	2,498 (3.5%)
Unknown	979 (19.7%)	865 (17.8%)	773 (15.5%)	682 (14%)	626 (12.6%)	630 (12.5%)	591 (11.4%)	586 (11.3%)	533 (10.1%)	497 (9.6%)	464 (9%)	461 (8.7%)	497 (9.2%)	479 (8.9%)	8,663 (12.1%)
**Smoking status**															
Active smoker	1,005 (20.2%)	1,071 (22%)	1,037 (20.8%)	1,028 (21%)	1,006 (20.3%)	1,040 (20.6%)	1,088 (21%)	1,087 (21%)	993 (18.8%)	1,078 (20.9%)	1,054 (20.5%)	1,053 (20%)	1,066 (19.8%)	1,093 (20.3%)	14,699 (20.5%)
Ex-smoker	2,183 (43.9%)	2,168 (44.6%)	2,299 (46%)	2,280 (46.6%)	2,433 (49.1%)	2,459 (48.8%)	2,466 (47.7%)	2,565 (49.6%)	2,762 (52.2%)	2,540 (49.2%)	2,585 (50.3%)	2,549 (48.3%)	2,646 (49.1%)	2,583 (47.9%)	34,518 (48.1%)
Non-smoker	1,575 (31.7%)	1,443 (29.7%)	1,501 (30%)	1,428 (29.2%)	1,402 (28.3%)	1,442 (28.6%)	1,518 (29.4%)	1,436 (27.8%)	1,438 (27.2%)	1,442 (27.9%)	1,423 (27.7%)	1,604 (30.4%)	1,596 (29.6%)	1,623 (30.1%)	20,871 (29.1%)
Unknown	205 (4.1%)	176 (3.6%)	159 (3.2%)	152 (3.1%)	110 (2.2%)	102 (2%)	100 (1.9%)	84 (1.6%)	103 (1.9%)	101 (2%)	78 (1.5%)	67 (1.3%)	79 (1.5%)	92 (1.7%)	1,608 (2.2%)
**Comorbidities**															
Respiratory Disease	905 (18.2%)	900 (18.5%)	955 (19.1%)	939 (19.2%)	1,016 (20.5%)	1,034 (20.5%)	1,138 (22%)	1,124 (21.7%)	1,183 (22.3%)	1,128 (21.9%)	1,140 (22.2%)	1,074 (20.4%)	1,179 (21.9%)	1,196 (22.2%)	14,911 (20.8%)
Dementia	264 (5.3%)	266 (5.5%)	265 (5.3%)	278 (5.7%)	288 (5.8%)	308 (6.1%)	280 (5.4%)	303 (5.9%)	310 (5.9%)	294 (5.7%)	318 (6.2%)	311 (5.9%)	292 (5.4%)	282 (5.2%)	4,059 (5.7%)
Chronic Kidney Disease (Stage 3+)	1,049 (21.1%)	1,074 (22.1%)	1,144 (22.9%)	1,126 (23%)	1,102 (22.3%)	1,085 (21.5%)	1,080 (20.9%)	1,045 (20.2%)	994 (18.8%)	924 (17.9%)	900 (17.5%)	834 (15.8%)	842 (15.6%)	789 (14.6%)	13,988 (19.5%)
Cancer	1,108 (22.3%)	1,112 (22.9%)	1,241 (24.8%)	1,227 (25.1%)	1,242 (25.1%)	1,356 (26.9%)	1,381 (26.7%)	1,367 (26.4%)	1,490 (28.1%)	1,445 (28%)	1,473 (28.7%)	1,507 (28.6%)	1,556 (28.9%)	1,541 (28.6%)	19,046 (26.6%)

Liver Disease	61 (1.2%)	70 (1.4%)	72 (1.4%)	79 (1.6%)	76 (1.5%)	83 (1.6%)	91 (1.8%)	91 (1.8%)	115 (2.2%)	142 (2.8%)	141 (2.7%)	149 (2.8%)	166 (3.1%)	169 (3.1%)	1,505 (2.1%)
Diabetes	894 (18%)	936 (19.3%)	1,045 (20.9%)	988 (20.2%)	1,110 (22.4%)	1,114 (22.1%)	1,155 (22.3%)	1,197 (23.1%)	1,249 (23.6%)	1,225 (23.7%)	1,258 (24.5%)	1,291 (24.5%)	1,332 (24.7%)	1,355 (25.1%)	16,149 (22.5%)
Heart Failure	409 (8.2%)	399 (8.2%)	394 (7.9%)	373 (7.6%)	394 (8%)	366 (7.3%)	391 (7.6%)	378 (7.3%)	402 (7.6%)	352 (6.8%)	352 (6.8%)	348 (6.6%)	347 (6.4%)	360 (6.7%)	5,265 (7.3%)
Atrial Fibrillation	871 (17.5%)	904 (18.6%)	932 (18.7%)	890 (18.2%)	965 (19.5%)	928 (18.4%)	960 (18.6%)	910 (17.6%)	998 (18.8%)	872 (16.9%)	875 (17%)	881 (16.7%)	855 (15.9%)	830 (15.4%)	12,671 (17.7%)
Hypertension	2,833 (57%)	2,764 (56.9%)	2,905 (58.1%)	2,844 (58.2%)	2,859 (57.7%)	2,872 (57%)	2,978 (57.6%)	2,989 (57.8%)	3,070 (58%)	2,883 (55.9%)	2,994 (58.2%)	2,934 (55.6%)	2,984 (55.4%)	3,020 (56%)	40,929 (57.1%)
Ischaemic Heart Disease	2,288 (46.1%)	2,301 (47.4%)	2,279 (45.6%)	2,350 (48.1%)	2,313 (46.7%)	2,336 (46.3%)	2,444 (47.3%)	2,358 (45.6%)	2,463 (46.5%)	2,331 (45.2%)	2,406 (46.8%)	2,453 (46.5%)	2,463 (45.7%)	2,520 (46.7%)	33,305 (46.5%)
Transient Ischaemic Attack	634 (12.8%)	633 (13%)	658 (13.2%)	592 (12.1%)	571 (11.5%)	608 (12.1%)	647 (12.5%)	612 (11.8%)	595 (11.2%)	595 (11.5%)	558 (10.9%)	531 (10.1%)	569 (10.6%)	522 (9.7%)	8,325 (11.6%)
Haemorrhage	795 (16%)	799 (16.4%)	868 (17.4%)	867 (17.7%)	917 (18.5%)	1,019 (20.2%)	1,025 (19.8%)	1,086 (21%)	1,133 (21.4%)	1,128 (21.9%)	1,134 (22.1%)	1,194 (22.6%)	1,226 (22.8%)	1,235 (22.9%)	14,426 (20.1%)
Significant Carotid Artery Disease	171 (3.4%)	166 (3.4%)	168 (3.4%)	162 (3.3%)	160 (3.2%)	169 (3.4%)	172 (3.3%)	193 (3.7%)	163 (3.1%)	219 (4.2%)	161 (3.1%)	155 (2.9%)	175 (3.2%)	186 (3.5%)	2,420 (3.4%)
Depression	1,022 (20.6%)	1,038 (21.4%)	1,094 (21.9%)	1,180 (24.1%)	1,173 (23.7%)	1,195 (23.7%)	1,328 (25.7%)	1,353 (26.2%)	1,415 (26.7%)	1,440 (27.9%)	1,441 (28.0%)	1,453 (27.6%)	1,577 (29.3%)	1,572 (29.2%)	18,281 (25.5%)

Supplementary Table 3. Cohort baseline characteristics at entry into the study, by stroke type, continued.

	Ischaemic	Haemorrhagic	Unspecified
	n = 44,909	n = 7,607	n = 19,180
**Sex**			
Male	23,269 (51.8%)	3,869 (50.9%)	9,383 (48.9%)
**Age (years)**			
Diagnosis	73.3 (13.6)	70.5 (15.7)	73.1 (14.0)
**WIMD Quintile**			
1 (Most deprived)	9,057 (20.2%)	1,383 (18.2%)	3,842 (20%)
2	9,421 (21%)	1,556 (20.5%)	3,987 (20.8%)
3	9,055 (20.2%)	1,570 (20.6%)	3,938 (20.5%)
4	8,700 (19.4%)	1,542 (20.3%)	3,826 (19.9%)
5 (Least deprived)	8,676 (19.3%)	1,556 (20.5%)	3,587 (18.7%)
**Rural/Urban classification**			
Rural town and fringe	5,948 (13.2%)	989 (13%)	2,682 (14%)
Rural town and fringe in a sparse setting	1,764 (3.9%)	354 (4.7%)	775 (4%)
Rural village and dispersed	2,742 (6.1%)	479 (6.3%)	1,219 (6.4%)
Rural village and dispersed in a sparse setting	3,434 (7.6%)	683 (9%)	1,356 (7.1%)
Urban city and town	30,131 (67.1%)	4,934 (64.9%)	12,809 (66.8%)
Urban city and town in a sparse setting	890 (2%)	168 (2.2%)	339 (1.8%)
**Ethnic group**			
Asian, Black, Mixed and other	706 (1.6%)	182 (2.4%)	318 (1.7%)
White	42,865 (95.4%)	7,139 (93.8%)	18,186 (94.8%)
Unknown	1,338 (3%)	286 (3.8%)	676 (3.5%)
**Weight category**			
Obese	12,553 (28%)	1,793 (23.6%)	5,443 (28.4%)

Overweight	14,199 (31.6%)	2,239 (29.4%)	6,018 (31.4%)
Normal weight	11,383 (25.3%)	2,122 (27.9%)	4,785 (24.9%)
Underweight	1,482 (3.3%)	329 (4.3%)	687 (3.6%)
Unknown	5,292 (11.8%)	1,124 (14.8%)	2,247 (11.7%)
**Smoking status**			
Active smoker	9,449 (21%)	1,385 (18.2%)	3,865 (20.2%)
Ex-smoker	21,655 (48.2%)	3,536 (46.5%)	9,327 (48.6%)
Non-smoker	12,852 (28.6%)	2,456 (32.3%)	5,563 (29%)
Unknown	953 (2.1%)	230 (3%)	425 (2.2%)
**Comorbidities**			
Respiratory Disease	9,259 (20.6%)	1,481 (19.5%)	4,171 (21.7%)
Dementia	2,194 (4.9%)	472 (6.2%)	1,393 (7.3%)
Chronic Kidney Disease (Stage 3+)	9,036 (20.1%)	1,167 (15.3%)	3,785 (19.7%)
Cancer	11,942 (26.6%)	2,107 (27.7%)	4,997 (26.1%)
Liver Disease	898 (2%)	222 (2.9%)	385 (2%)
Diabetes	10,611 (23.6%)	1,273 (16.7%)	4,265 (22.2%)
Heart Failure	3,476 (7.7%)	421 (5.5%)	1,368 (7.1%)
Atrial Fibrillation	8,228 (18.3%)	1,300 (17.1%)	3,143 (16.4%)
Hypertension	26,217 (58.4%)	3,863 (50.8%)	10,849 (56.6%)
Ischaemic Heart Disease	21,266 (47.4%)	3,141 (41.3%)	8,898 (46.4%)
Transient Ischaemic Attack	5,115 (11.4%)	542 (7.1%)	2,668 (13.9%)
Haemorrhage	7,642 (17%)	3,328 (43.7%)	3,456 (18%)
Significant Carotid Artery Disease	2,205 (4.9%)	22 (0.3%)	193 (1%)
Depression	11,082 (24.7%)	1,873 (24.6%)	5,326 (27.8%)

Supplementary Table 4. Cohort baseline characteristics at entry into the study, by care setting of diagnosis, continued.

	Primary care	Secondary care
	n = 21,305	n = 50,391
**Sex**		
Male	11,154 (52.4%)	25,367 (50.3%)
**Age (years)**		
Diagnosis	70.8 (13.8)	73.9 (13.9)
**WIMD Quintile**		
1 (Most deprived)	4,135 (19.4%)	10,147 (20.1%)
2	4,149 (19.5%)	10,815 (21.5%)
3	4,309 (20.2%)	10,254 (20.3%)
4	4,409 (20.7%)	9,659 (19.2%)
5 (Least deprived)	4,303 (20.2%)	9,516 (18.9%)
**Rural/Urban classification**		
Rural town and fringe	2,686 (12.6%)	6,933 (13.8%)
Rural town and fringe in a sparse setting	898 (4.2%)	1,995 (4%)
Rural village and dispersed	1,374 (6.4%)	3,066 (6.1%)
Rural village and dispersed in a sparse setting	1,716 (8.1%)	3,757 (7.5%)
Urban city and town	14,172 (66.5%)	33,702 (66.9%)
Urban city and town in a sparse setting	459 (2.2%)	938 (1.9%)
**Ethnic group**		
Asian, Black, Mixed and other	386 (1.8%)	820 (1.6%)
White	20,486 (96.2%)	47,704 (94.7%)
Unknown	433 (2%)	1,867 (3.7%)
**Weight category**		

Obese	6,146 (28.8%)	13,643 (27.1%)
Overweight	6,984 (32.8%)	15,472 (30.7%)
Normal weight	5,322 (25%)	12,968 (25.7%)
Underweight	611 (2.9%)	1,887 (3.7%)
Unknown	2,242 (10.5%)	6,421 (12.7%)
**Smoking status**		
Active smoker	4,670 (21.9%)	10,029 (19.9%)
Ex-smoker	10,170 (47.7%)	24,348 (48.3%)
Non-smoker	6,152 (28.9%)	14,719 (29.2%)
Unknown	313 (1.5%)	1,295 (2.6%)
**Comorbidities**		
Respiratory Disease	4,544 (21.3%)	10,367 (20.6%)
Dementia	1,113 (5.2%)	2,946 (5.8%)
Chronic Kidney Disease (Stage 3+)	3,524 (16.5%)	10,464 (20.8%)
Cancer	5,403 (25.4%)	13,643 (27.1%)
Liver Disease	486 (2.3%)	1,019 (2%)
Diabetes	4,558 (21.4%)	11,591 (23%)
Heart Failure	1,292 (6.1%)	3,973 (7.9%)
Atrial Fibrillation	3,044 (14.3%)	9,627 (19.1%)
Hypertension	11,850 (55.6%)	29,079 (57.7%)
Ischaemic Heart Disease	9,741 (45.7%)	23,564 (46.8%)
Transient Ischaemic Attack	2,885 (13.5%)	5,440 (10.8%)
Haemorrhage	5,045 (23.7%)	9,381 (18.6%)
Significant Carotid Artery Disease	2,252 (10.6%)	168 (0.3%)
Depression	6,013 (28.2%)	12,268 (24.3%)

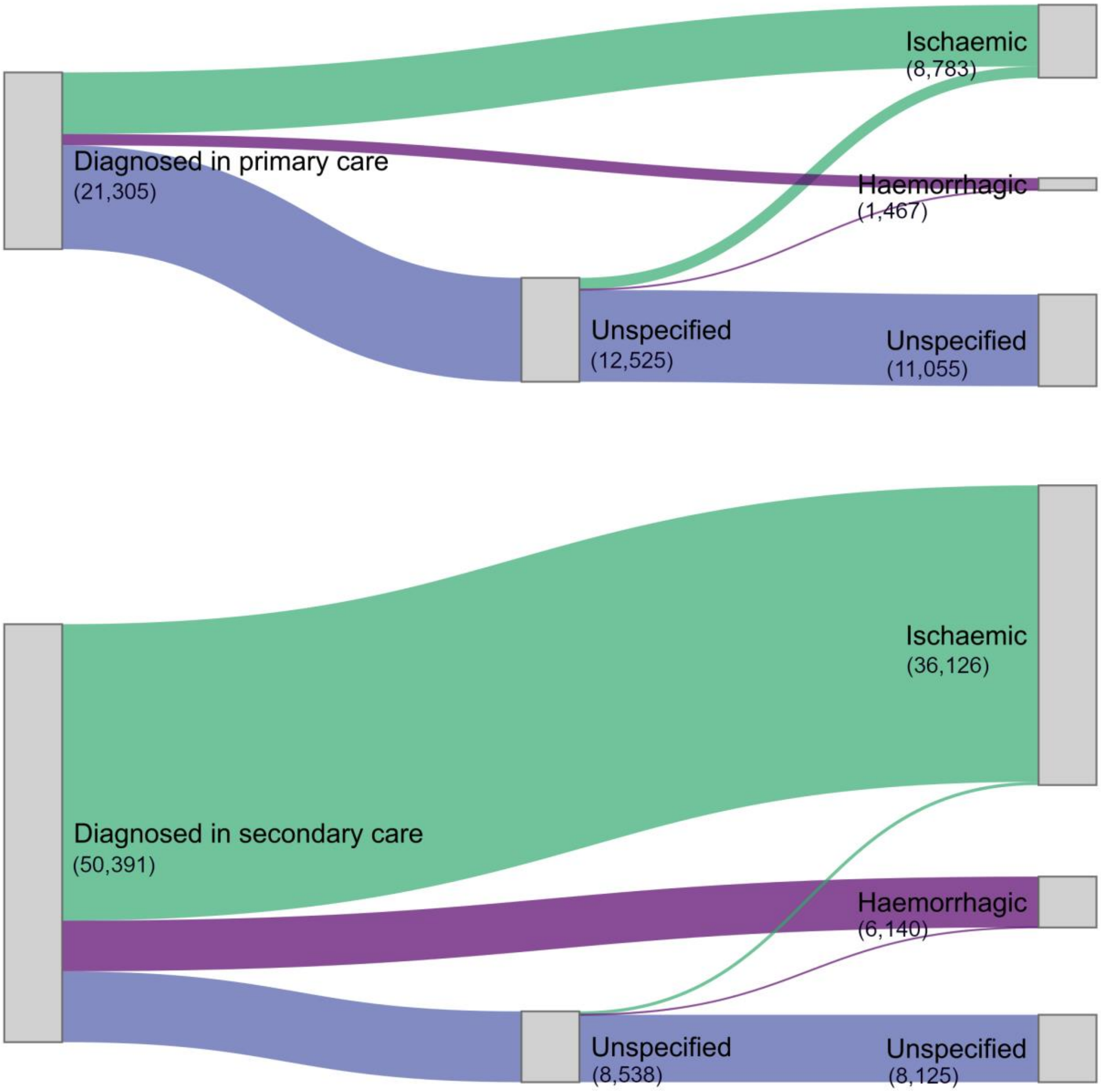

Supplementary Figure 1. Care setting of diagnosis and reclassification of stroke type following first record

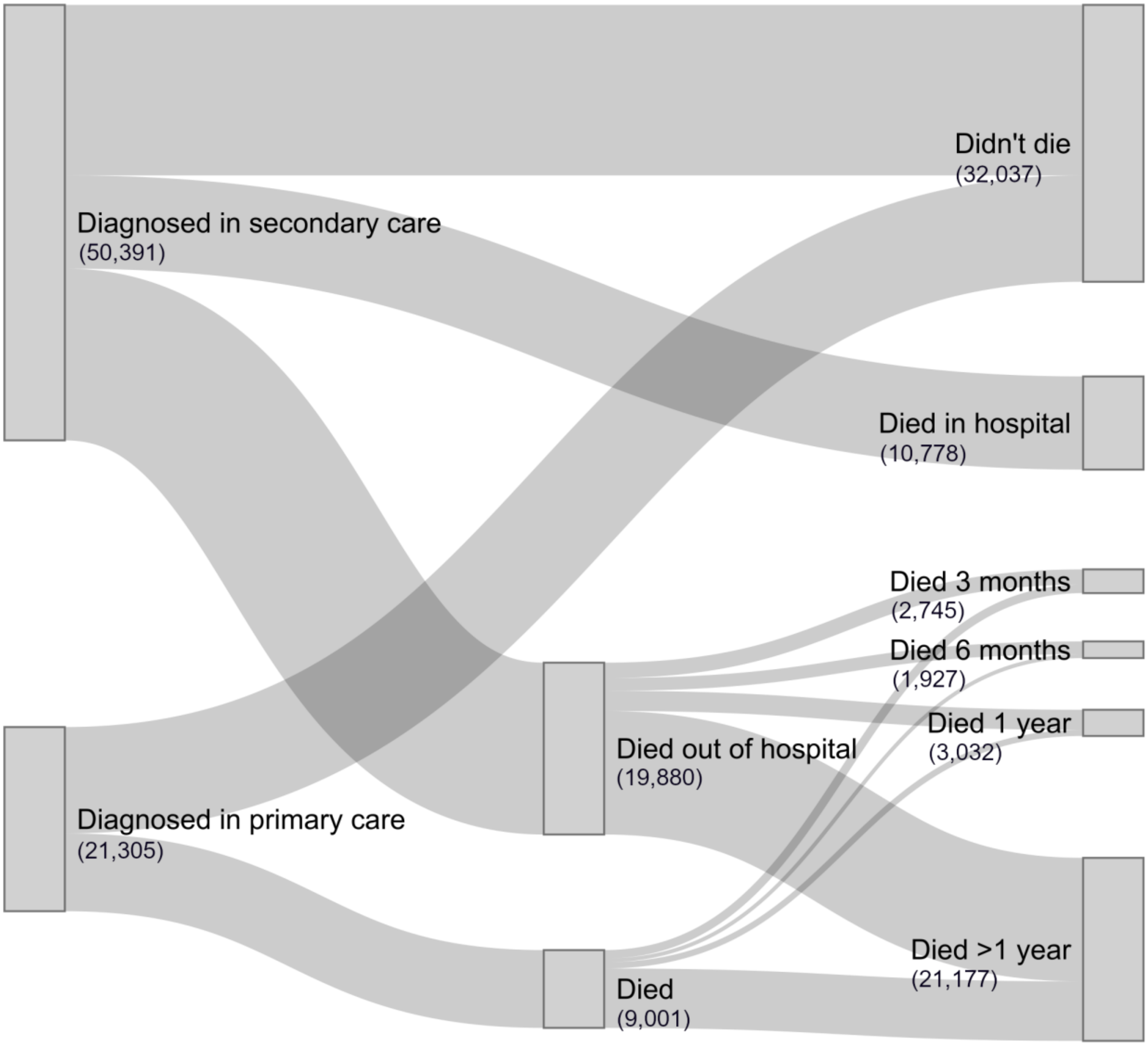

Supplementary Figure 2. Care setting of diagnosis and death outcomes in the year following diagnosis.
